# Regulatory region mutations of *TERT*, *PLEKHS1* and *GPR126* genes as urinary biomarkers in upper tract urothelial carcinomas

**DOI:** 10.7150/jca.56779

**Published:** 2021-05-05

**Authors:** Xiangling Xing, Xiaotian Yuan, Tiantian Liu, Mingkai Dai, Yidong Fan, Cheng Liu, Klas Strååt, Magnus Björkholm, Dawei Xu

**Affiliations:** 1 Department of Medicine, Division of Hematology, Bioclinicum and Center for Molecular Medicine (CMM), Karolinsk Institutet and Karolinska University Hospital Solna, SE-17176 Stockholm, Sweden.; 2 Pathology Department, School of Basic Medical Science, Shandong University, Jinan, PR China.; 3 Central Research Laboratory, the Second Hospital, Cheeloo College of Medicine, Shandong University, Jinan, 250033, PR China.; 4 Department of Urology, Qilu Hospital, Cheeloo College of Medicine, Shandong University, Jinan, 250012, PR China.; 5 Department of Urology, Peking University Third Hospital, Beijing, PR China.

**Keywords:** GPR126, PLEKHS1, TERT, Urinary biomarker, UBC, UTUC

## Abstract

**Background:** The hotspot regulatory region mutations of the TERT, PLEKHS1 and GPR126 genes have been shown to occur frequently in urothelial bladder carcinoma (UBC). However, it is currently unclear whether these mutations are all present in upper tract urothelial carcinomas (UTUC) including renal pelvic carcinoma (RPC) and ureter carcinoma (UC), although TERT promoter mutations were previously observed in these malignancies.

**Methods:** The hotspot mutations of TERT and PLEKHS1 promoters and GPR126 intron 6 (enhancer) in tumors derived from 164 patients with UTUC were determined using Sanger sequencing, and the obtained results were further compared with the mutation frequency in 106 UBCs. The mutations were also assessed in urine from patients with UTUC and UBC.

**Results:** The mutation frequencies in UTUC tumors were 28%, 5.8% and 11% for TERT and PLEKHS1 promoters and GPR126 intron 6, respectively, which were lower than those (44.3%, 26.4%, and 31.4%, respectively) in UBCs. The total frequencies for the presence of any of these mutations were 50.8% and 34.4% for RPCs and UCs, respectively. All these mutated DNA sequences were detectable in urine from both UTUC and UBC patients and disappeared rapidly in most patients after surgery.

**Conclusions:** This proof-of-concept study demonstrates that the hotspot mutations in the TERT, PLEKHS1 and GPR126 non-coding regions are present in UTUCs, and that urinary assays of these mutated sequences serve as potential biomarkers for UTUC diagnostics and disease monitoring.

## Introduction

Urothelial carcinomas originate from the urothelium or belong to transitional cell carcinomas, and consist primarily of upper tract urothelial carcinomas (UTUCs) and urothelial bladder carcinomas (UBCs) 1. UTUCs include renal pelvic carcinomas (RPCs) and ureter carcinomas (UCs), and their incidence is much lower compared to UBCs, but has increased gradually over the past two decades, especially in East Asia including China 1, 2. The majority of UTUCs have become invasive or metastatic when diagnosed, mainly due to the lack of early clinical symptoms and of specific diagnostic approaches 3-7. Currently, UTUC diagnosis is mainly based on imaging and endoscopy, while non-invasive methods such as bladder urine cytology, a standard tool for UBC diagnostics, has very limited sensitivity in UTUCs 5, 7. In order to improve clinical management and outcomes of UTUCs, oncologists and scientists are striving to search for reliable disease bio-markers and to develop accurate diagnostic tools.

The activation of telomerase, a RNA-dependent DNA polymerase lengthening telomeres, is essential to the malignant transformation of human cells 8, 9. Recently, the recurrent mutation in the proximal promoter of the *telomerase reverse transcriptase* (*TERT*) gene, which encodes the catalytic, rate-limiting component of the telomerase complex, has been identified to drive telomerase activation in many human malignancies including UTUCs and UBCs 10-15. Two hotspot mutations in the TERT promoter so-called C228T and C250T create *de novo* ETS binding motifs, thereby activating TERT transcription and telomerase 16, 17. We previously observed that the frequencies of TERT promoter mutations were 48% and 19% in primary RPC and UC tumors, respectively 12. Moreover, the mutated TERT promoter DNA was detectable in patients' urine, and thus served as a urine-based diagnostic marker 12. However, as described above, more than 50% of RPC and 80% UC tumors carry a wild type (wt) TERT promoter, and hence, other urinary biomarkers are required for these patients.

Recent studies have identified the hotspot mutations in the PLEKHS1 promoter and GPR126 intron 6 in UBCs 18-20. In UBC primary tumors, mutation rates were approximately 30 - 48% and 50% for the PLEKHS1 promoter and GPR126 intron 6, respectively 19, 20. Both UTUCs and UBCs originate from the urothelium and share certain molecular events in the pathogenesis, however, their molecular alterations may be distinct, or differ substantially due to different anatomical locations or other unknown reasons 1, 4. It is currently unclear whether these mutations also occur in RPCs and UCs, and if so, whether they overlap with TERT promoter mutations, or whether they can be used as biomarkers in patients with a wt TERT promoter. In the present study, we simultaneously determined the mutations in the TERT and PLEKHS1 promoters and GPR126 intron 6 in UTUC tumors, compared the results with UBCs, and finally evaluated their potential clinical implications.

## Patients and methods

### UTUC and UBC patients and tumor specimens

The study was conducted on 164 patients with UTUC (68 RPCs and 96 UCs) and 106 patients with UBC who underwent surgery at Shandong University Qilu Hospital and Second Hospital, China. UTUC and UBC were diagnosed according to the criteria of the World Health Organization (WHO) 21. Patients' characteristics are summarized in Tables 1 and 2, which include sex, age at diagnosis, tumor size, pathology stage, clinical stage/metastases. The specimens were collected after surgical treatment and kept frozen at -80 ºC. All samples were collected with written informed consent and the study was approved by the Shandong University Second Hospital ethics committee.

### Voided urine samples from UTUC and UBC patients

Spontaneously voided urine was collected from 89 patients including 13 UTUCs and 76 UBCs prior to surgical treatment. In 20 of these 89 patients, urine was also consecutively obtained one week post-operation. Fifty ml of urine were centrifuged and cell pellets were kept at -80 ºC until use.

### DNA extraction and sequencing

Genomic DNA was extracted from frozen tumor tissue samples and urine pellets using QIAGEN DNA extraction kits. The mutations of TERT and PLEKHS1 promoters and GPR126 intron 6 were analyzed using Sanger sequencing as described 22, 23. The following primers were used for Sanger sequencing: The TERT promoter: 5'-CAC CCG TCC TGC CCC TTC ACC TT-3' (forward) and 5'-GGC TTC CCA CGT GCG CAG CAG GA-3' (reverse); the PLEKHS1 promoter: 5'-GAA TCC TCG GGA CAA GGC ACT-3' (forward) and 5'-GTC AGT CCT ATT TCC CTC TGA CT-3' (reverse); GPR126 intron 6: 5'-CCA AGG AGA TTT ATG ATG GAG CAA-3' (forward) and 5'-GCA GAG AGA GAT GGC CTA AAC A-3' (reverse). The mutations were verified by sequencing from both directions.

### Statistical analyses

Unless stated, all statistical analyses were performed using IBM SPSS Statistics version 24 (IBM, Armonk, NY). Differences in the TERT and PLEKHS1 promoter or GPR126 intron 6 mutation frequencies in relation to sex, TNM stage, pathology stage, tumor size, number of tumors, recurrence, were determined using Chi-Square or Fisher's Exact Test. Mann-Whitney U Test was used to analyze differences in age between the mutation-positive (mt) and negative (wt) groups. P values of < 0.05 were considered as statistically significant. Sensitivity is defined as numbers of true mutation-positive samples/numbers of mutation positive samples detected from tumor tissues. Specificity is defined as numbers of true mutation-negative samples/numbers of mutation-negative samples in tumor tissues. Accuracy is defined as numbers of correct assessments/numbers of all samples.

To visualise proportions of different mutations in both tumor and urine samples, annotated pie chart and permutation test was performed using SPICE 6.0 (NIH). Patient samples with any missing mutation information were excluded from the SPICE analysis.

## Results

### The mutation frequency of TERT and PLEKHS1 promoters and GPR126 intron 6 in UTUC tumors

**Figure 1:**
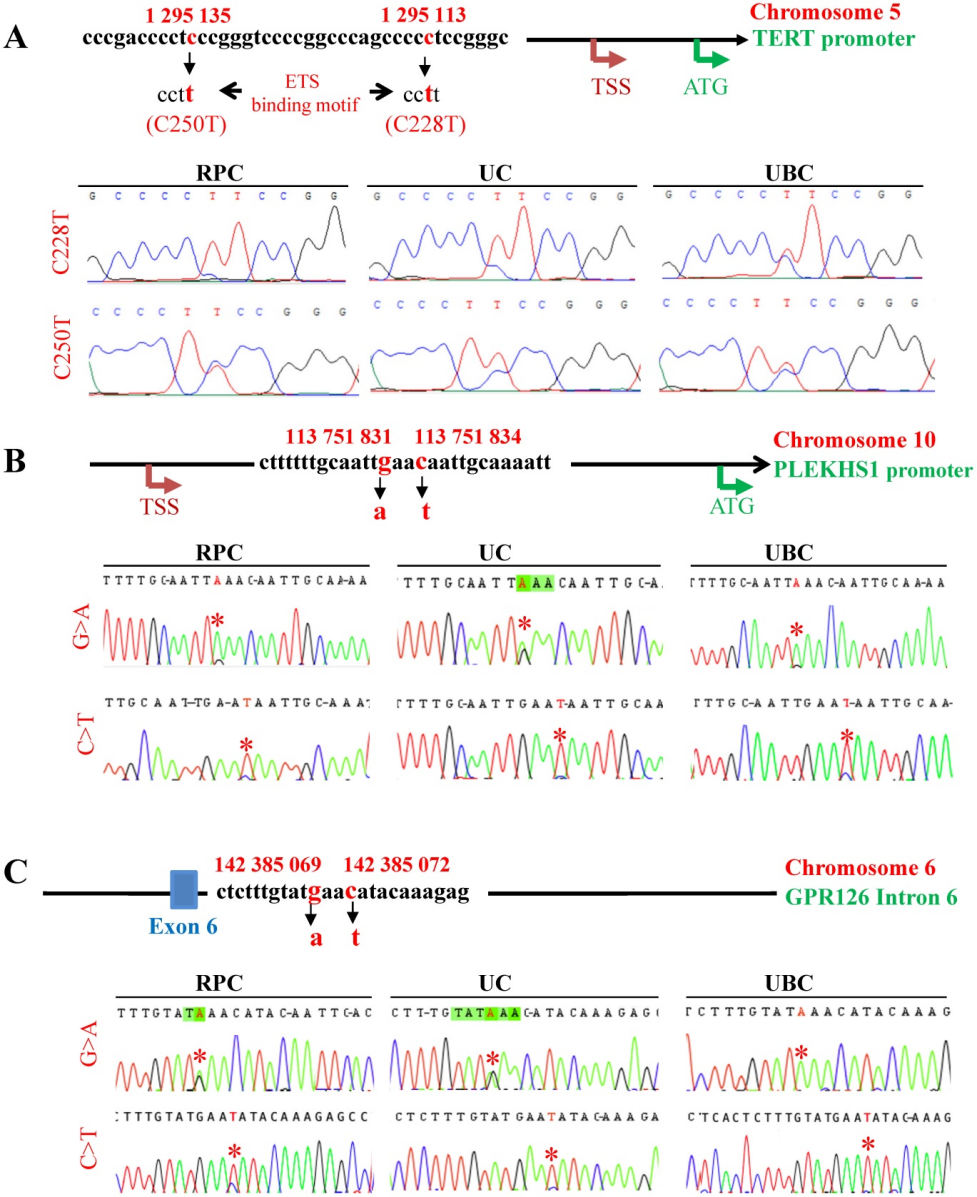
** The mutations of the TERT and PLEKHS1 promoters and GPR126 intron 6 in UTUC and UBC tumors**. Tumor genomic DNA derived from patients with UTUC and UBC was analyzed for TERT and PLEKHS1 promoter and GPR126 intron 6 mutations using Sanger sequencing. TSS: Transcription start site; RPC: Renal pelvic carcinoma; UC: Ureter carcinoma. (A) TERT promoter mutations. Top panel: The schematic of the TERT proximal promoter and mutation locations. The *TERT* gene is localized on chromosome 5, and two hotspot mutations, which result from a cytidine-to-thymidine (C>T) dipyrimidine transition at 1 295 113 (assembly to GRCh38.p13, or 1 295 228 assembly GRCh37.p13), and 1 295 135 (assembly to GRCh38.p13, or 1 295 250 assembly to GRCh37.p13), are named C228T and C250T, respectively. Both mutations create *de novo* ETS1 binding motifs through which TERT transcription is activated. Bottom panel: The representative chromatograms show C228T and C250T mutations (Marked with *) in UTUC and UBC tumors, respectively. (**B**) PLEKHS1 promoter mutations. Top panel: The schematic of hotspot mutations in the PLEKHS1 promoter on chromosome 10. Two mutation hotspots are indicated (G>A 113 751 831) and (C>T 113 751 834), according to the GRCh38.p13 assembling. In addition, G>C at 113 751 831 or C>G at 113 751 834 may occur occasionally. These two mutations are flanked by stretches of 10 bp on both sides that are palindromic to each other. Bottom panel: The representative chromatograms show two different mutations of the PLEKHS1 promoter (Marked with *) in UTUC and UBC tumors, respectively. (**C**) GPR126 intron 6 mutations. Top panel: The schematic of hotspot mutations in the intron 6 of the *GPR126* gene on chromosome 10. Two hotspot mutations are indicated (G>A 142 385 069) and (C>T 142 385 072), respectively (assembly to GRCh38.p13). In addition, G>C at 142 385 069 or C>G at 142 385 072 may occur in a small fraction of tumors. There exist similar palindromic sequences seen in the mutated PLEKHS1 promoter, which are flanked by a stretch of 11 bps on both sides. Bottom panel: The representative chromatograms show two different mutations of the GPR126 intron 6 (Marked with *) in UTUC and UBC tumors, respectively.

Mutational status in TERT and PLEKHS1 promoters and GPR126 intron 6 was analyzed in tumor DNA derived from 164 patients with UTUC using Sanger sequencing (Fig. 1). A total of 164 patients in the present cohort were evaluable for the TERT promoter status: the tumors carrying a wt and mutated (mt) TERT promoter were 118 (72%) and 46 (28%), respectively (Fig. 1A). The C228T mutation was predominant (37/46, 80%) while C250T occurred in only 9/46 (20%) of tumors (Table 1). For the PLEKHS1 promoter and GPR126 intron sequencing, 155 of 164 tumors were successfully studied (Table 1). Nine of 155 tumors (5.8%) harboured the mt PLEKHS1 promoter, among which 6 (6/64, 9.4%) were RPCs while 3 (3/91, 3.3%) were UCs (Fig. 1B) (Tables 2 and 3). In these tumors bearing the mt PLEKHS1 promoter, 6/9 had C>T, while 3 of them were G>A mutations. The GPR intron 6 mutation was identified in 17 of 155 tumors (11%), which included 5 RPCs (5/62, 8.1%) and 12 UCs (12/93, 12.9%) (Tables 2 and 3). These results thus demonstrate that all these mutations occur in UTUCs with different frequencies.

**Figure 2:**
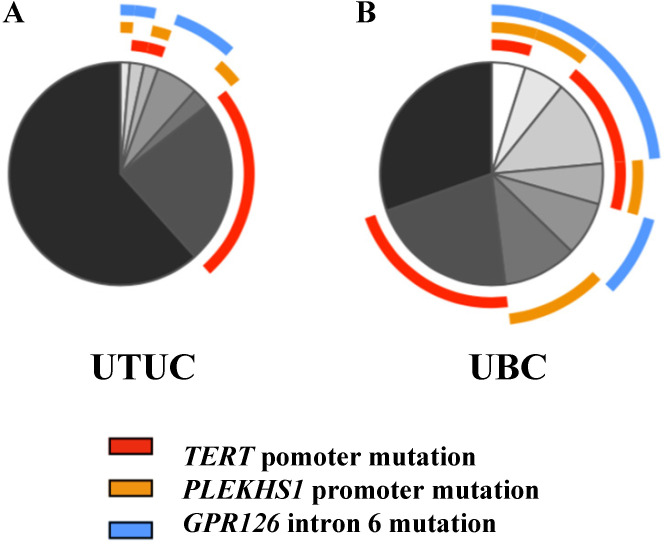
** Proportions of different mutations in UTUC and UBC cohorts.** (A) Annotated pie chart for mutation frequencies in UTUC cohort (n = 146). (B) Annotated pie chart for mutation frequencies in UBC cohort (n = 102).

The mutations of the TERT and PLEKHS1 promoters and GPR126 intron 6 occurred in most UTUC tumors separately whereby only less than 3% of the cohort had co-existing mutations (Fig. 2), and there was no association between these three mutations. In a small fraction of tumors, TERT promoter mutations co-existed with either PLEKHS1 promoter (3 tumors) or GPR126 intron 6 (4 tumors) mutations, whereas the PLEKHS1 promoter and GPR126 mutations occurred together in 2 UTUC tumors. The total frequencies for the presence of any of these mutations were 50.8% and 34.4% for RPCs and UCs, respectively.

### Comparison of mutation frequencies between UTUCs and UBCs

The data above indicate much lower frequencies in PLEKHS1 and GPR126 mutations in UTUCs than observed in UBCs, as recently reported 18-20. However, such a difference may be also due to the differences in ethnicity, geography or environment and carcinogen exposure 11. To elucidate this question, we further analyzed 106 UBC patients diagnosed and treated at the same hospitals, both cohorts of UBC and UTUC patients being Han Chinese. The TERT and PLEKHS1 promoter and GPR126 intron 6 mutations in this cohort of UBC patients were 47/106 (44.3%), 28/106 (26.4%), and 32/102 (31.4%), respectively, which were indeed significantly higher than those in the present cohort of UTUCs (Fig. 2 and Table 4). The total frequencies for the presence of any of these mutations were 67.9%, much higher than that in UTUCs. Five UBC tumors carried all three mutations, while the co-occurrence of the mt TERT promoter together with the mt PLEKHS1 promoter and GPR126 intron 6 were found in 6 and 13 UBC tumors, respectively.

### Detection of TERT and PLEKHS1 promoter and GPR126 intron 6 mutations in urine from patients with UTUC and UBC

**Figure 3:**
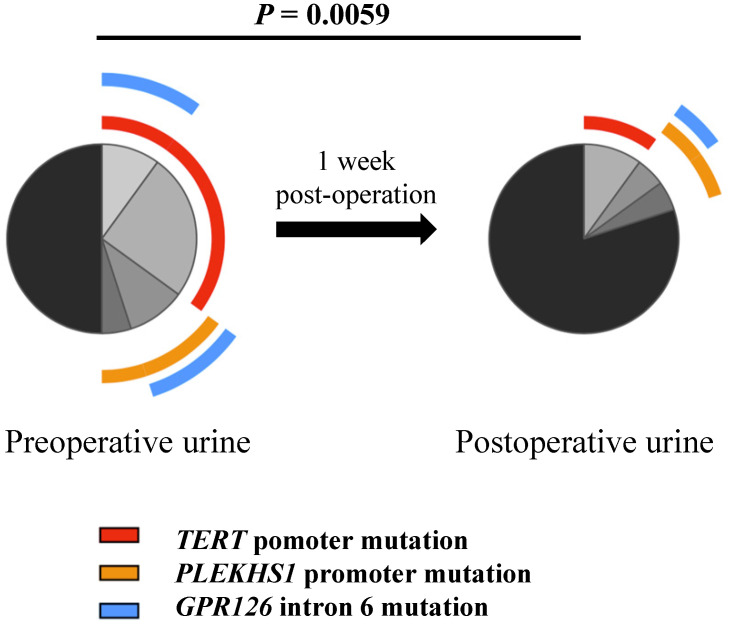
** Differential proportions of mutated fragments detected in pre-operative and post-operative urine samples.** Permutation test was used to test for significance in comparison between pre-operative and post-operative urine samples (n = 20).

We and others previously showed that the mutated TERT promoter sequence was detectable in urine derived from UTUC and UBC patients, and served as a urinary diagnostic biomarker 10-12, 15, 24. Here we further sought to determine whether all these mutated fragments could be found in the urine from UTUC and UBC patients. The urinary samples from 13 UTUC patients and 76 UBC patients were collected before surgery and Sanger sequencing was carried out. As shown in Table 5, the mutated TERT and PLEKHS1 promoters and GPR126 intron 6 were indeed detected in patient urine, although the sensitivity for Sanger sequencing was only 60%. Nonetheless, a high consistency between tumors and urine reflects satisfactory specificity (Table 5). In addition, the urinary samples collected one week post-operation surgery were available in 20 of these patients and 10 of them were positive for mutated TERT and/or PLEKHS1 promoters or GPR126 intron 6 in their urine before surgery. In general, we observed a significant reduction in the frequencies of mutated fragments from post-operative samples (Fig. 3). The sequencing analysis showed that the mutated DNA fragments in urine disappeared in 6 of 10 patients after tumor removal. Their rapid disappearance indicates that they may be also reliable biomarkers for disease surveillance of UTUCs and UBCs.

### Association of the mutations with clinical variables in UTUCs and UBCs

We finally determined a potential relationship between the detected mutations and additional clinical variables in patients with UTUC and UBC (Tables 1 and 4). For TERT promoter mutations, a significantly higher frequency was seen in older UC patients (*P* = 0.005), but there were no associations between the TERT promoter mutation and TNM or pathological stages, tumor sizes, metastasis or recurrence in UC or RPC (Tables 2 and 3). In UBCs, the presence of mutated TERT promoter mutations was significantly associated with disease recurrence and male sex (*P* = 0.021 and 0.041, respectively) (Table 4). For PLEKHS1 promoter and GPR126 intron 6 mutations, no associations with any of tested clinic-pathological variables were observed in UTUCs (Table 2 and 3), while the GPR126 mutations were associated with small tumors in patients with UBCs (< 3 cm vs ≥ 3 cm, *P* = 0.006) (Table 4).

## Discussion

The recurrent TERT promoter mutations have been previously identified in UTUCs and UBCs, and the mutant DNA sequences are detectable in patient urine, thereby serving as a urinary biomarker for UTUC and UBC diagnostics 10-12, 24, 25. Such an approach doesn't work for tumors carrying a wt TERT promoter, including most UTUC patients who lack TERT promoter mutations. In the present study, we comprehensively analyzed the non-coding mutations in TERT and PLEKHS1 promoters and GPR126 intron 6 in UTUCs, and demonstrated that these mutations all occurred in primary UTUC tumors.

The early detection of UTUCs is currently challenging, and it is thus more demanding to develop reliable methods for this purpose 1, 10, 12. Urine-based tests are a non-invasive diagnostic tool for UTUC, but require specific biomarkers. Because normal urothelial cells lack mutations in the TERT and PLEKHS1 promoters or GPR126 intron 6, they should be ideal urinary biomarkers for UTUC diagnosis. Indeed, these mutant DNA sequences were found in urine from patients with mutation-bearing tumors, which indicate that these mutation assays have potentially clinical implications in UTUCs. In fact, the mutated TERT promoter has been previously evaluated as a urinary biomarker for UTUC or UBC diagnostics and disease surveillance, and the promising results have been obtained 10-12, 24, 25. Interestingly, Hosen et al found that the TERT promoter mutation could be present in urine as early as 10 years before a clinical diagnosis of UBC 25. Like UBCs, UTUCs also originate from the urothelium and belong to transitional cell carcinomas, and it is thus plausible that this genetic alteration occurs much earlier in UBUCs, too. Moreover, PLEKHS1 promoter and GPR126 intron 6 mutations may similarly take place in the precursor lesion during UTUC pathogenesis. If confirmed, all these three mutations will be perfect urinary biomarkers to screen the general population with UTUC/UBC risk factors.

In the present study, we determined the mutations of TERT and PLEKHS1 promoters and GPR126 intron 6 using Sanger sequencing, a gold standard for the identification of mutant targets. Based on our previous report, the threshold sensitivity of Sanger sequencing is at least 10% of mutant TERT promoter-containing tumor DNA 10. However, voided urine contains both normal and malignant cells, and if the later fraction is too small, it might be difficult to catch the mutant target by Sanger sequencing. Such a scenario is expected with small either primary or recurring tumors producing few exfoliated tumor cells. Indeed, the present analysis results of patient urine only had less than 50% of sensitivity for Sanger sequencing. Thus, it is strongly motivated to develop more sensitive assays to detect minor proportions of mutant alleles present in bulk urinary DNA. For instance, castPCR and digital droplet PCR have been developed to analyze the mutated TERT promoter in urine derived from UTUC and UBC patients with much higher sensitivity and specificity 10, 26. These same techniques should also be established for PLEKHS1 promoter and GPR126 mutation assays. In addition, next generation sequencing has also emerged as a powerful tool to simultaneously analyze multiple targets including TERT and PLEKHS1 promoter and GPR126 intron 6 mutations.

The mutations of these three genes were also evaluated for their clinic-pathological association in UTUCs. Neither PLEKHS1 promoter nor GPR126 intron 6 mutations were significantly correlated with clinic-pathological variables, which was consistent with other reports on UBCs 19, 20. Nonetheless, PLEKHS1 over-expression has been shown to predict shorter survival in UBC patients, while higher GPR126 levels seem to be associated with favourable outcomes 19, 20. In accordance, we found that PLEKHS1 drove AKT hyperactivity, promoted metastasis and was associated with shorter survival in thyroid cancer 23. For TERT promoter mutations, we observed a strongly positive correlation between the mutation and disease recurrence in UBCs. The TERT promoter mutation creates *de novo* ETS binding motifs to activate the *TERT* transcription and telomerase, thereby empowering cancer cells with a proliferation advantage 17. Moreover, TERT displays multiple activities and is capable of protecting cancer cells from apoptosis stimulated by various insults and facilitating invasion or metastasis by inducing epithelial-mesenchymal transition or other pro-metastatic cascades 9, 27-29.

## Conclusions

TERT and PLEKHS1 promoter and GPR126 intron mutations occur in UTUCs, although the frequencies are lower than those in UBC. Importantly, these mutated DNA fragments are detectable in patient urine. Because the mutations are absent in normal urothelial cells, they may serve as specific biomarkers for UTUC diagnostics and disease monitoring, which is supported by our proof-concept study. Liquid biopsies including non-invasive urinary tests for cancer diagnosis and surveillance have become more and more attractive, and toward this purpose, reliable biomarkers together with highly sensitive, specific methods such as ddPCR are required. It should also be pointed out that the mutations of TERT and PLEKHS1 promoters and GPR126 intron 6 do not occur in all patients, and these mutation assays are not applicable for patients who lack these genetic alterations. Therefore, we still need to look for new biomarkers to include remaining patients. In the meanwhile, advanced technologies including next generation sequencing should be useful by analyzing multi-targets simultaneously. Finally, the close association between TERT promoter mutations and recurrent disease may be valuable in UTUC/UBC prognostication, treatment decision and follow-up designs. Taken together, the present findings are implicated in the diagnostics and management of patients with UTUC and UBC.

## Figures and Tables

**Table 1:** Clinic-pathological variables and association with TERT and PLEKHS1 promoter and GPR126 intron 6 mutations in UTUC patients

	TERT promoter (N = 164)			PLEKHS1 promoter (N = 155)			GPR126 intron 6 (N = 155)		
	wt	mt	*P*	wt	mt	*P*	wt	mt	*P*
	118/164 (72%)	46/164 (28%)		146/155 (94.2%)	9/155 (5.8%)		138/155 (89%)	17/155 (11%)	
Age (Median, years)	67 (32 - 83)	65 (36 - 87)	0.643	67 (36 - 87)	72 (58 - 78)	0.157	65 (36 - 87)	68 (32 - 81)	0.337
**Gender**			0.356			1.000			0.351
Female	45	14		50	3		49	8	
Male	73	32		96	6		89	9	
**TNM stage**			0.429			0.685			0.765
pTa + pT1	30	9		34	1		35	3	
≥ pT2	88	37		112	8		103	14	
**Pathology stage**			0.664			0.135			0.549
G1	19	9		27	0		24	1	
G2	9	5		12	2		12	1	
G3	90	32		107	7		102	15	
**Tumor size**			0.731			0.077			0.743
< 3 cm	46	18		58	1		54	7	
≥ 3 cm	54	24		68	8		65	7	
**LN metastasis**			0.285			1.000			1.000
No	109	45		137	9		130	16	
Yes	9	1		9	0		8	1	
**Distant metastasis**			0.135			0.262			0.445
No	116	43		142	8		134	16	
Yes	2	3		4	1		4	1	
**Recurrence**			0.313			1.000			0.375
No	116	44		142	9		135	16	
Yes	2	2		4	0		3	1	

LN: Lymph node; mt: mutant; wt: wild type; UTUC: Upper track urothelial carcinoma.

**Table 2:** Clinic-pathological variables and association with TERT and PLEKHS1 promoter and GPR126 intron 6 mutations in RPC patients

	TERT promoter (N = 68)			PLEKHS1 promoter (N = 64)			GPR126 intron 6 (N = 62)		
	wt	mt	*P*	wt	mt	*P*	wt	mt	*P*
	41/68 (60.3%)	27/68 (39.7%)		58/64 (90.6%)	6/64 (9.4%)		57/62 (91.9%)	5/62 (8.1%)	
Age (Median, years)	67 (40 - 80)	64 (36 - 77)	0.156	63 (36 - 80)	72.5 (58 - 78)	0.095	63 (36 - 80)	69 (64 - 73)	0.174
**Gender**			0.286			0.671			1.000
Female	19	9		22	3		24	2	
Male	22	18		36	3		33	3	
**TNM stage**			0.787			0.584			0.574
pTa + pT1	8	6		12	0		13	0	
≥ pT2	33	21		46	6		44	5	
**Pathology stage**			0.487			0.312			0.699
G1	7	6		12	0		10	0	
G2	2	3		4	1		4	0	
G3	32	18		42	5		43	5	
**Tumor size**			0.140			0.176			0.648
< 3 cm	10	11		17	0		17	2	
≥ 3 cm	30	15		39	6		38	3	
**LN metastasis**			0.514			1.000			1.000
No	39	27		56	6		55	5	
Yes	2	0		2	0		2	0	
**Distant metastasis**			0.154			0.180			1.000
No	41	25		57	5		55	5	
Yes	0	2		1	1		2	0	

LN: Lymph node; mt: mutant; wt: wild type; RPC: Renal pelvic carcinoma.

**Table 3:** Clinic-pathological variables and association with TERT and PLEKHS1 promoter and GPR126 intron 6 mutations in UC patients

	TERT promoter (N = 96)			PLEKHS1 promoter (N = 91)			GPR126 intron 6 (N = 93)		
	wt	mt	*P*	wt	mt	*P*	wt	mt	*P*
	77/96 (80.2%)	19/96 (19.8%)		88/91 (96.7%)	3/91 (3.3%)		81/93 (87.1%)	12/93 (12.9%)	
Age (Median, years)	67 (32 - 83)	74 (55 - 87)	**0.005**	67.5 (41 - 87)	71 (64 - 76)	0.519	67 (41 - 87)	67.5 ( 32 - 81)	0.909
**Gender**			0.534			0.550			0.205
Female	26	5		28	0		25	6	
Male	51	14		60	3		56	6	
**TNM stage**			0.383			1.000			1.000
pTa + pT1	22	3		22	1		22	3	
≥ pT2	55	16		66	2		59	9	
**Pathology stage**			1.000			0.333			0.871
G1	12	3		15	0		14	1	
G2	7	2		8	1		8	1	
G3	58	14		65	2		59	10	
**Tumor size**			0.244			0.571			1.000
< 3 cm	36	7		41	1		37	5	
≥ 3 cm	24	9		29	2		27	4	
**LN metastasis**			1.000			1.000			1.000
No	70	18		81	3		75	11	
Yes	7	1		7	0		6	1	
**Distant metastasis**			0.488			1.000			0.343
No	75	18		85	3		79	11	
Yes	2	1		3	0		2	1	
**Recurrence**			0.174			1.000			0.430
No	75	17		84	3		78	11	
Yes	2	2		4	0		3	1	

LN: Lymph node; mt: mutant; wt: wild type; UC: Ureter carcinoma. *P* values in bold indicate significant differences.

**Table 4:** Clinic-pathological variables and association with TERT and PLEKHS1 promoter and GPR126 intron 6 mutations in UBC patients

	TERT promoter (N = 106)			PLEKHS1 promoter (N = 106)			GPR126 intron 6 (N = 102)		
	wt	mt	*P*	wt	mt	*P*	wt	mt	*P*
	59/106 (55.7%)	47/106 (44.3%)		78/106 (73.6%)	28/106 (26.4%)		70/102 (68.6%)	32/102 (31.4%)	
Age (Median, years)	66 (36 - 87)	64 (44 - 89)	0.055	64.5 (36 - 86)	67 (43 - 89)	0.253	65 (36 - 89)	64 (50 - 87)	0.751
**Gender**			0.041			0.512			0.304
Male	47	44		68	23		58	29	
Female	12	3		10	5		12	3	
**TNM stage**			0.802			0.337			0.765
pTa + pT1	44	33		54	23		50	24	
≥ pT2	15	10		20	5		17	7	
**Pathology stage**			0.345			0.885			1.000
G1	6	2		6	2		5	2	
G2	19	20		30	9		25	11	
G3	34	25		42	17		40	19	
**Tumor size**			0.242			0.496			0.006
< 3 cm	23	24		36	11		25	21	
≥ 3 cm	35	23		41	17		44	11	
**Number of tumors**			0.955			1.000			0.552
Single	33	27		44	16		41	17	
Multiple	25	20		33	12		28	15	
**Recurrence**			0.021			0.762			0.697
No	52	33		62	23		57	25	
Yes	7	14		16	5		13	7	

LN: Lymph node; mt: mutant; wt: wild type; UBC: Urothelial bladder carcinoma. *P* values in red indicate significant differences.

**Table 5:** Consistency of TERT/PLEKHS1/GPR126 mutations between tumor tissues and urine samples from patients with UTUC and UBC as determined using Sanger sequencing

Urine samples	Tissue samples				
	MT (n)	WT (n)	Sensitivity (%)	Specificity (%)	Accuracy (%)
MT (n)	36	3	61%	90%	70.7%
WT (n)	23	27			
**Total**	59	30			

MT: Mutant; WT: Wild type; UBC: Urothelial bladder carcinoma; UTUC: Upper track urothelial carcinoma.

## Abbreviations

RPC: renal pelvic carcinoma
TERT: Telomerase reverse transcriptase
UBC: urothelial bladder carcinoma
UC: Ureter carcinoma
UTUC: upper track urothelial carcinoma
